# Front-Line Therapy for Metastatic Renal Cell Carcinoma: A Perspective on the Current Algorithm and Future Directions

**DOI:** 10.3390/cancers14092049

**Published:** 2022-04-19

**Authors:** Ameish Govindarajan, Daniela V. Castro, Zeynep B. Zengin, Sabrina K. Salgia, Jalen Patel, Sumanta K. Pal

**Affiliations:** City of Hope Comprehensive Cancer Center, Duarte, CA 91010, USA; agovindarajan@coh.org (A.G.); dacastro@coh.org (D.V.C.); zzengin@coh.org (Z.B.Z.); sabrina.salgia@gmail.com (S.K.S.); jalenpatel827@gmail.com (J.P.)

**Keywords:** renal cell carcinoma, targeted therapy, immunotherapy

## Abstract

**Simple Summary:**

The treatment landscape of metastatic renal cell carcinoma has changed dramatically over the course of the last decade. The use of doublet immunotherapy or targeted therapy/immunotherapy regimens has become the standard of care. Herein, we outline the important elements and considerations in the treatment of renal cell carcinoma and explore prospective treatment options within this setting.

**Abstract:**

Over the last decade, the treatment paradigm of metastatic renal cell carcinoma has rapidly evolved, with notable changes in the front-line setting. Combination therapies involving the use of either doublet therapy with immune checkpoint inhibitors or combination VEGFR-directed therapies with immune checkpoint inhibitors have significantly improved clinical outcomes, including prolonged overall survival and durable response to treatment. We aim to highlight the Food and Drug Administration-approved front-line therapy options, the navigation of treatment selection, and the future directions of metastatic renal cell carcinoma therapies.

## 1. Introduction

The treatment of metastatic renal cell carcinoma (mRCC) represents one of the great achievements of modern oncology. Over the past two decades, the prognosis of this disease has improved drastically. The first Food and Drug Administration (FDA)-approved regimen for mRCC was high-dose interleukin-2 which, along with the regimen including interferon-α, represented the standard of care; however, the median survival rates associated with these treatments were 12 months and 13 months, respectively [1]. These cytokine-based therapies were supplanted a decade later by targeted therapies that modulated signaling through the vascular endothelial growth factor receptor (VEGFR), leading to a doubling of median survival [1,2,3,4]. More recently, the introduction of checkpoint inhibitors (CPIs) has led to a further improvement in clinical outcomes and disease prognosis, mirroring the results in multiple other cancer types [5,6,7,8,9,10].

**Figure 1:**
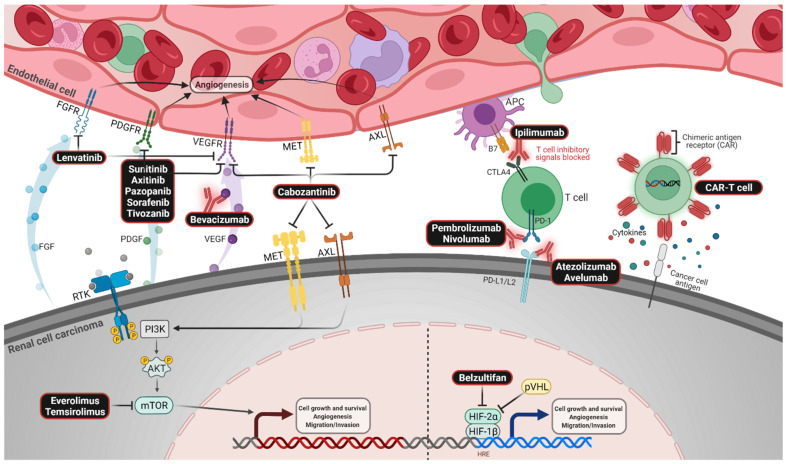
Mechanism of action of experimental and Food and Drug Administration-approved drugs. In this figure, only the major molecules in each pathway are depicted. Created with BioRender.com. Abbreviations: FGF—fibroblast growth factor; FGFR—FGF receptor; PDGF—platelet-derived growth factor receptor; PDGFR—PDGF receptor; VEGF—vascular endothelial growth factor; VEGFR—VEGF receptor; MET—hepatocyte growth factor receptor; RTK—receptor tyrosine kinase; PI3K—phosphoinositide 3-kinase; AKT—protein kinase B; mTOR—mechanistic target of rapamycin complex; APC—antigen-presenting cell; PD-1—programmed cell death protein; PD-L1—PD-1 ligand 1; PD-L2—PD-1 ligand 2; HIF—hypoxia-inducible factor; HRE—hypoxia response element; pVHL—von Hippel–Lindau protein.

This review will summarize the progress made in the first-line treatment setting of mRCC, with a primary focus on results pertaining to patients diagnosed with the most common variant, clear cell histology (75–80%) [11]. In this paper, we will provide insight into the most recent trial data evaluating combination VEGFR-directed therapy with CPIs, as well as dual CPI therapy, and outline gaps in the existing literature and avenues for future research. The mechanisms of action of FDA-approved drugs in the treatment of mRCC are outlined in Figure 1.

## 2. Dual CPI Therapy

The combination of nivolumab with ipilimumab has demonstrated activity in multiple disease settings, including melanoma, non-small cell lung cancer, unresectable mesothelioma, hepatocellular carcinoma, and an array of other diseases [5,6,7,8]. In the setting of mRCC, among the first dual CPIs to be investigated was the phase I dose escalation study of nivolumab and ipilimumab (CheckMate 016) [12]. The first arm of this trial included a higher dose of nivolumab (3 mg/kg) with a lower dose of ipilimumab (1 mg/kg; N3I1), while the second arm included a dosage of nivolumab at 1 mg/kg and ipilimumab at 3 mg/kg (N1I3). The study showed that while overall response rate (40.4%) and 2-year overall survival rate (67 to 70%) were not statistically different between dose regimens, grade 3–4 treatment-related adverse event frequency was significantly higher in N1I3 (61.7%) compared to the N3I1 (38.3%) arm. As a result, a regimen of nivolumab at 3 mg/kg with ipilimumab at 1 mg/kg was further assessed in the phase III CheckMate 214 study.

In CheckMate 214, patients were randomized in a 1:1 fashion to receive either nivolumab/ipilimumab or sunitinib at a standard dose of 50 mg daily, with a 4 weeks on, 2 weeks off schedule [9]. A similar dosing strategy was utilized for sunitinib in all the studies discussed herein, and thus will not be detailed further. Eligibility was largely comparable to other front-line studies, enrolling treatment-naïve patients diagnosed with clear cell histology (a sarcomatoid component was permitted). The initial co-primary endpoints of the study were overall survival (OS), response rate, and progression-free survival (PFS), with emphasis given to outcomes among patients with intermediate- and poor-risk disease, as defined by the International mRCC Database Consortium (IMDC) criteria [13]. The CheckMate 214 study possesses the longest follow-up of any doublet therapy trial reported to date. At a median follow-up of 67.7 months, the study has continued to meet the primary endpoint of improved OS compared to sunitinib, demonstrating a median overall survival of 47.0 months vs. 26.6 months for the intermediate/poor-risk population (HR 0.68; 95% CI, 0.58–0.81; *p* < 0.0001) [10]. Median PFS was also superior in the intermediate/poor-risk population (11.6 months vs. 8.3 months; HR 0.73; 95% CI, 0.61–0.87; *p* = 0.0004), as was response rate (42.0% vs. 27.0%). Notably, however, contrasting results were observed in the favorable-risk population. In this sub-population, 5-year PFS probabilities appeared to favor sunitinib (12.4 months vs. 28.9 months; HR 1.60; 95% CI, 1.13–2.26; *p* = 0.0073). The most common toxicities associated with nivolumab/ipilimumab were fatigue (37%), pruritus (28%), and diarrhea (27%) [9]. Grade 3–4 treatment-related adverse events (TRAEs) were less common in the dual CPI arm compared to sunitinib (47.9% vs. 64.1%), with corticosteroids employed to manage TRAEs in 29.1% of the nivolumab/ipilimumab population [14]. 

## 3. CPI with VEGFR-Directed Therapy

To date, there have been four combination CPI/VEGFR-directed therapies approved by the FDA for the treatment of mRCC. Preceding the four trials that led to these approvals, one phase III study evaluated the combination of the VEGF-directed monoclonal antibody bevacizumab with the programmed-death-ligand 1 (PD-L1) inhibitor atezolizumab. The phase III IMmotion151 trial randomized patients to receive bevacizumab/atezolizumab or sunitinib monotherapy [15]. In this trial, no significant differences in OS were observed between the treatment arms. The median OS for intention-to-treat (ITT) patients treated with bevacizumab/atezolizumab was 36.1 months compared to 35.3 months for sunitinib (HR 0.91; 95% CI, 0.76–1.08; *p* = 0.27) [16], although bevacizumab/atezolizumab was favored over sunitinib among those with PD-L1+ with regard to median PFS (11.2 months vs. 7.7 months; HR 0.74; 95% CI, 0.57–0.96; *p* = 0.02). A the study did meet the primary endpoint of investigator-assessed PFS, the regimen did not ultimately receive FDA approval [15]. 

The results of the first two phase III studies evaluating VEGFR–tyrosine kinase inhibitors (VEGFR-TKIs) with CPIs were presented simultaneously. These studies both used a backbone of axitinib, but paired it with two distinct CPIs, avelumab (in the JAVELIN Renal 101 trial) and pembrolizumab (in the KEYNOTE-426 trial) [17,18]. The studies evaluated slightly different endpoints—in the JAVELIN Renal 101 trial, the co-primary endpoints of PFS and OS in the PD-L1+ population were evaluated, while in the KEYNOTE-426 study, co-primary endpoints of PFS and OS in the ITT population were evaluated. In the JAVELIN Renal 101 trial, a greater response rate was noted among those treated with axitinib plus avelumab, as compared to sunitinib in the PD-L1+ sub-population (55.2% vs. 25.5%) [17,19]. A significant benefit in PFS was also observed among these patients (13.8 months vs. 7.2 months; HR 0.61; 95% CI, 0.47–0.79; *p* < 0.001); however, OS in the axitinib plus avelumab group has yet to be reached (42.2 months—not estimable) as compared to the sunitinib group (37.8 months; 31.4 months—not estimable) [19]. Although this regimen has received FDA approval, because of a lack of a defined OS advantage, it does not currently represent a preferred front-line treatment option [17,19]. The most common toxicities observed within the axitinib plus avelumab arm were noted to be diarrhea, hypertension, and fatigue [17]. 

In contrast, the KEYNOTE-426 trial demonstrated benefits with axitinib and pembrolizumab across multiple endpoints, including PFS in the ITT population (15.1 months vs. 11.1 months; HR 0.69; 95% CI, 0.57–0.84; *p* < 0.001), OS at 12 months and 18 months (89.9% vs. 78.3% and 82.3% vs. 72.1%, respectively) and overall response rate (59.3% vs. 35.7%), as compared to sunitinib [18]. Notably however, long-term follow-up (42.8 months) has shown some diminution in the OS benefit associated with axitinib/pembrolizumab, with the initial hazard ratio of 0.53 (95% CI, 0.38–0.74) now increased to 0.73 (95% CI, 0.60–0.88 [20]. It is possible that this increase may reflect the efficacy of second-line therapies in the control arm, including those receiving approved CPIs such as nivolumab. The most frequently observed toxicities with the combination of axitinib and pembrolizumab included diarrhea, hypertension, fatigue, and hypothyroidism [18]. A higher incidence of grade 3 and 4 hepatic adverse events were reported among the former combination arm as compared to either axitinib or pembrolizumab monotherapy, with the most common hepatotoxicities being elevations in alanine aminotransferase and aspartate aminotransferase [21]. 

Two phase III trials have been presented following KEYNOTE-426: the CheckMate 9ER trial and the CLEAR trial. The CheckMate 9ER study evaluated the multikinase inhibitor cabozantinib in combination with nivolumab, again comparing this combination to sunitinib [22]. The dose of cabozantinib evaluated in this trial was 40 mg, lower than the approved dose of cabozantinib in second-line therapy (60 mg). A significant advantage in median PFS was observed with cabozantinib/nivolumab compared to sunitinib (16.6 months vs. 8.3 months; HR 0.51; 95% CI, 0.41–0.64; *p* < 0.001) [22]. Superior outcomes were also noted in the secondary endpoint of response rate (55.7% vs. 27.1%; *p* < 0.001); however, median OS has not yet been reached in any of the cabozantinib/nivolumab subgroups. The most common toxicities noted with cabozantinib/nivolumab were diarrhea, palmar–palmar erythrodysesthesia, and hypertension, seen in 63.8%, 40.0%, and 34.7% of patients. 

The CLEAR trial employed a multi-comparison schema, comparing lenvatinib (a multikinase inhibitor) with everolimus (a mammalian target of rapamycin (mTOR) inhibitor) and lenvatinib with pembrolizumab to the sunitinib-based control arm [23]. Just as in CheckMate 9ER, different permutations of lenvatinib dosing were explored. The lenvatinib/everolimus arm received the FDA-approved dosing strategy in the second-line setting, with lenvatinib at 18 mg daily and everolimus at 5 mg daily. In contrast, the lenvatinib/pembrolizumab arm received a higher dose of lenvatinib (20 mg). Superior PFS was observed relative to sunitinib in both the lenvatinib/everolimus (14.7 months vs. 9.2 months; HR 0.65; 95% CI, 0.53–0.80; *p* < 0.001) and lenvatinib/pembrolizumab treatment arms (23.9 months vs. 9.2 months; HR 0.39; 95% CI, 0.32–0.49; *p* < 0.001) [23]. The overall response rates for lenvatinib/pembrolizumab, lenvatinib/everolimus, and sunitinib were reported as 71.0%, 55.3%, and 36.1%, respectively; OS has yet to be reached but was significantly improved with lenvatinib/pembrolizumab versus sunitinib. The most common toxicities noted among the lenvatinib/pembrolizumab treatment arm were diarrhea, hypertension, hypothyroidism, and decreased appetite.

Importantly, while the reviewed treatment classes possess different pharmacologic targets, their combination can result in overlapping or additive toxicities. As such, the treating physician must be aware of toxicities that may arise from individual agents as well as combination therapies when selecting front-line and subsequent therapies, and their severity and frequency should be carefully monitored and managed. There are several strategies that may aid in optimizing toxicity management. For example, the primary driver of a concerning toxicity may be discerned by stopping the TKI and evaluating the extent of toxicity after several half-lives of the drug. If toxicities persist beyond this period, CPI-related toxicity should be suspected. 

## 4. Selecting Front-Line Therapy: General Considerations

Each of the phase III trials discussed above represent a major milestone in mRCC therapy, as seen in Table 1. A challenge for the practicing medical oncologist is to incorporate these complex datasets into a uniform algorithm for therapy. With the exception of the JAVELIN Renal 101, each of these studies have suggested an improvement in OS with CPI-based combinations as compared to sunitinib, leaving little rationale for the use of axitinib/avelumab in this setting. Notably however, there are currently neither head-to-head trials comparing these FDA-approved treatment combinations, nor biomarkers available to help guide treatment choices.

In order to gain insight into this issue, Aeppli and colleagues examined factors associated with treatment decision-making in the first-line setting among cancer experts [30]. The following diverse criteria were most crucial: IMDC risk stratification; performance status; contraindications to CPIs; compulsion to obtain an effective rapid response; and PD-L1 expression. IMDC risk stratification and contraindications to CPIs are currently the most important factors in guiding treatment choice among favorable- and intermediate-/poor-risk groups, with 7 out of 11 experts employing a regimen of axitinib plus pembrolizumab to treat those in the IMDC favorable-risk group without contraindications to CPIs. In the IMDC intermediate/poor-risk group, on the other hand, the preferred treatment regimen was nivolumab/ipilimumab for patients without contraindications to CPIs. Of note, PD-L1 expression was not heavily favored (2 out of the 11 experts); however, in the event of high PD-L1 expression, the treatment choice was nivolumab/ipilimumab in the IMDC favorable-risk group, given no contraindications to CPIs. Notably, more recent FDA-approved combination therapies, such as cabozantinib plus nivolumab and lenvatinib plus pembrolizumab, were not included in this study, as these regimens had not been approved at the time of data collection, thus limiting the breadth of this study. 

A recent systematic review sought to further guide treatment selection by comparing PFS, OS, overall response rate (ORR), and TRAEs across six randomized phase III clinical trials: CheckMate 214, IMmotion 151, JAVELIN Renal 101, KEYNOTE-426, CheckMate 9ER, and the CLEAR trial [31]. The subsequent meta-analysis, with a total of 5121 patients, established TKI/CPI combinations as possessing the highest likelihood of conferring superior PFS and OS as compared to doublet CPIs, and thus recommended the use of TKI/CPI combinations regardless of IMDC risk group and PD-L1 expression status. Cabozantinib/nivolumab, followed by lenvatinib/pembrolizumab, were associated with the highest likelihood of conferring superior OS, while nivolumab plus ipilimumab had the highest complete response rates and the lowest likelihood of grade 3–4 TRAEs. However, these TRAEs were more permanent and irreversible compared to grade 3–4 toxicities associated with TKI/CPI combinations.

Similar results were reported in a further meta-analysis comparing the OS, PFS, ORR and treatment-related AEs of four phase III randomized clinical trials in the setting of treatment-naïve patients with mRCC (CheckMate 214, KEYNOTE-426, CheckMate 9ER and CLEAR) [32]. The combination of lenvatinib/pembrolizumab followed by cabozantinib/nivolumab conferred the greatest PFS and ORR, while cabozantinib/nivolumab was superior in OS with maximal complete response, partial response, and stable disease. Nivolumab/ipilimumab had the lowest likelihood of grade 3–4 TRAEs. 

In general, therefore, combination VEGFR-TKI/CPI therapies appear to provide the most notable improvements in clinical endpoints as compared to dual CPI therapy with nivolumab/ipilimumab. Thus, while this latter combination certainly has merits (e.g., superior quality of life (QoL) compared to sunitinib, and the extension of treatment-free intervals), our current practice is to offer VEGFR-TKI/CPI therapy as a first-line agent, except in special circumstances [9]. For example, patients being evaluated for surgery can commence treatment with nivolumab/ipilimumab immediately, without concern for any surgical delays. In contrast, VEGFR-TKI-based therapy must be withheld before and for several weeks after surgery, thus delaying effective systemic therapy [33]. Finally, dual CPI therapy remains the most appropriate regimen when there are contraindications to VEGFR-TKI agents, including uncontrolled hypertension or congestive heart failure. In choosing between the three approved VEGFR-TKI/CPI combinations (axitinib/pembrolizumab, cabozantinib/nivolumab, lenvatinib/pembrolizumab), a review of traditional clinical trial endpoints can help guide treatment selection. Our current practice has shifted away from the use of axitinib/pembrolizumab, given its more modest gains in PFS, while available data pertaining to QoL suggests no advantage with this regimen over sunitinib [34]. The same is true for lenvatinib/pembrolizumab, perhaps due to the higher dose of lenvatinib used in the front-line setting (20 mg) as compared to the refractory setting (18 mg) [23]. In contrast, data from the CheckMate 9ER study suggests that, with cabozantinib/nivolumab, QoL improves across several scales (National Comprehensive Cancer Network 19-item Function Assessment of Cancer Therapy-Kidney Symptom Index [FKSI-19]; 9-item subset of disease-related symptoms (FKSI-DRS)). Moreover, from our experience, the lower dose of cabozantinib used in the front-line setting versus the refractory setting (40 mg vs. 60 mg) does yield a perceptible decrease in toxicity burden [22]. Further targeted assessments of QoL may provide additional insight into varying toxicities associated within these different VEGFR-TKI/CPI regimens, and thus provide further guidance to clinicians. 

Finally, there are several ongoing investigations concerning the potential role of biomarkers in guiding treatment selection. PD-L1 status has been the most extensively studied biomarker in past trials of mRCC; however, its predictive value appears limited and current data shows that CPIs improve OS irrespective of PD-L1 status [35]. It is worthy of note that eligibility has not been traditionally restricted based on PD-L1 expression, nor have assays been standardized. For example, both CheckMate 214 and CheckMate 9ER used the Dako PD-L1 IHC 28-8 pharmDx assay (Agilent Technologies, Santa Clara, CA, USA), while the KEYNOTE-426 and CLEAR trials used the PD-L1 IHC 22C3 pharmDx assay (Agilent Technologies, Santa Clara, CA, USA), and JAVELIN Renal 101 used the Ventana PD-L1 (SP263) assay (Ventana Medical Systems, Tucson, AZ, USA) to measure expression [9,17,18,22,23]. This lack of standardization in the measurement of PD-L1 expression poses a challenge in effectively comparing outcomes across trials or drawing broader conclusions. 

## 5. Special Circumstances

Although this review and the aforementioned clinical trials have focused on clear cell mRCC, approximately 20% of patients possess non-clear cell mRCC. For these patients and their providers there are few established standards to guide treatment [36]. Papillary histology represents the most prevalent subset of non-clear cell disease, constituting approximately 15% of cases of RCC [37]. Genomic insights into this disease suggest that it is driven by alterations in the MET proto-oncogene [38]. The recent SWOG 1500 study randomized patients to either sunitinib or one of three MET inhibitors (cabozantinib, savolitinib, or crizotinib) [39]. The study, which included 147 eligible patients with 0-1 prior lines of treatment, met its primary endpoint, demonstrating an improvement in PFS with cabozantinib over sunitinib (9.0 months vs. 5.6 months; HR 0.60; 95% CI, 0.37–0.97; one-sided *p* = 0.019). Furthermore, the response rate of cabozantinib versus sunitinib was reported as 23% and 4%, respectively. As such, cabozantinib has been adopted across several guidelines as the preferred therapy for papillary mRCC [40]. Importantly however, this study included heterogenous subtypes of metastatic papillary RCC (PRCC type 1 and type 2) and was unable to restrict eligibility to patients with documented alterations in the *MET* protooncogene. A further question remains as to whether the combination of cabozantinib and a CPI may yield even better outcomes among this cohort. In two separate studies, the combinations of (1) cabozantinib with atezolizumab and (2) cabozantinib with nivolumab in papillary mRCC patients yielded response rates of 47% and 48%, respectively [41,42]. The upcoming randomized SWOG 2200 study will compare cabozantinib to cabozantinib with atezolizumab in patients with papillary mRCC and may help to definitively answer this question.

The treatment of other non-clear cell diseases also remains challenging, with such variants as chromophobe histology accounting for only 5% of cases of RCC [36]. In a small series of nine patients enrolled in a prospective study, four patients achieved a partial response with lenvatinib and everolimus [43]. The aforementioned experiences exploring cabozantinib with either atezolizumab or nivolumab yielded far lower responses. For even rarer histologies (e.g., translocation RCC, collecting duct RCC, and others), the benefit of combination therapies is largely anecdotal. 

Further research is also needed to help define optimal treatment strategies for patients that progress beyond adjuvant therapy. Adjuvant pembrolizumab was recently approved on the basis of the KEYNOTE-564 trial, which randomized patients with high-risk localized RCC to either pembrolizumab or placebo [44]. The study met its primary endpoint of disease-free survival (DFS), demonstrating a 2-year DFS of 77.3% with pembrolizumab as compared to 68.1% with the placebo (HR 0.68; 95% CI, 0.53–0.87; *p* = 0.002). As medical oncologists begin to use adjuvant pembrolizumab among their patients with localized disease, the benefit of front-line CPI may be called into question. The CONTACT-03 study, currently in progress, is seeking to compare cabozantinib alone to cabozantinib with atezolizumab [45], with a primary study population including patients with prior CPI use, either in the first- or second-line setting, but also allowing for patients with adjuvant CPI exposure. It is hoped that this trial will provide important guidance as to the optimal treatment approach in this population.

A final special circumstance concerns the role of cytoreductive nephrectomy (CN) in the setting of de novo mRCC. Although there remains some evidence supporting the role of CN, especially in palliative settings for symptomatic patients with abdominal pain, paraneoplastic syndromes, or to relieve gross hematuria, prospective data has failed to demonstrate its utility, and post-operative complications remain a concern (4% major complications; 22% complications of any kind) [46,47]. Further clarification will likely come from ongoing trials, such as PROBE (NCT04510597) and NORDIC-SUN (NCT03977571); however, existing retrospective data supports co-treatment with CN and CPI therapy. Prospective data is warranted and CN should only be considered in selected cases [48,49].

## 6. Conclusions

The many doublet therapies described in this manuscript have had a dramatic impact on therapy, but it is possible that the future landscape of therapy for mRCC may include triplet therapies (Table 2). The COSMIC-313 study, comparing nivolumab/ipilimumab (standard of care control arm) to cabozantinib/nivolumab/ipilimumab, has recently completed accrual [50]. It is hoped that this study will provide guidance for daily clinical practice and highlight the fact that combination therapy is now the standard of care among these patients. 

Beyond the scope of this manuscript there are many novel agents in early development for the treatment of mRCC. For example, agents that inhibit the hypoxia-inducible factor (HIF), such as belzutifan, have shown early promise in refractory mRCC and have been transformative in the context of patients with VHL syndrome. In a Phase II, single-group trial, the primary endpoint of objective response rate was reported to be 49% (95% CI, 36.0–62.0) within a cohort of RCC patients who had VHL disease, with a median follow-up time of 21.8 months [51]. Notably, within the same subset of RCC patients, no progression of disease was observed, and approximately half exhibited a partial response to treatment. This study led to the FDA approval of belzutifan in 2021 [52]. Belzutifan is now being explored in a variety of settings in the context of mRCC, including a large front-line trial comparing the combination of pembrolizumab with belzutifan and lenvatinib, the combination of pembrolizumab/quavonlimab with lenvatinib, and combination pembrolizumab and lenvatinib therapy [53]. There is also growing interest in cytokine-directed treatments in the front-line setting, with cutting-edge approaches targeting the IL-2 receptor pathway with a prodrug, bempegaldesleukin. For instance, PIVOT-09 (NCT03729245), a randomized phase III, open-label trial, is currently comparing bempegaldesleukin/nivolumab to either sunitinib or cabozantinib in treatment-naïve patients [54]. A further novel approach involves the utilization of vaccines in conjunction with combination therapies. One randomized phase II, open-label trial (NCT04203901) is comparing the use of CMN-001, an autologous, tumor antigen-loaded dendritic cell (immunotherapy), in combination with first-line nivolumab/ipilimumab followed by second-line lenvatinib/everolimus, to standard first-line nivolumab/ipilimumab and second-line lenvatinib/everolimus in advanced RCC [55]. These ongoing clinical trials, along with others, will help guide existing efforts to expand the treatment selection across all histological subtypes of RCC (Table 2).

Combination therapy approaches have become the mainstay of treatment in mRCC. Given their success, it is possible that other classes of therapy (bispecific antibodies or cellular therapies) may follow a similar trajectory. Since the advent of cytokines in the treatment of mRCC, the prognosis for advanced disease has improved dramatically, with a now growing focus on various genomic signatures and potential biomarkers that may help stratify patients and improve clinical outcomes further. Precision medicine, and the refined process of treatment selection, will rely on further investigation and trials of different treatment combinations head-to-head. The future holds promise, as clinical trials and novel studies will help to drive the development of an evidence-based and comprehensive treatment algorithm and, in turn, improve outcomes among patients with mRCC.

## Figures and Tables

**Table 1:** Combination Approaches in First-Line Metastatic Renal Cell Carcinoma.

Trial Name		CheckMate 214 [9,10]		KEYNOTE-426 [18,20]		CheckMate 9ER [22]		CLEAR [23]			JAVELIN Renal 101 [17,19,24]	
Treatment arm		Ipilimumab + Nivolumab *n* = 550	Sunitinib *n* = 546	Axitinib + Pembrolizumab *n* = 432	Sunitinib *n* = 429	Cabozantinib + Nivolumab *n* = 323	Sunitinib *n* = 328	Lenvatinib + Pembrolizumab *n* = 355	Lenvatinib + Everolimus *n* = 357	Sunitinib *n* = 357	Axitinib + Avelumab *n* = 270	Sunitinib *n* = 290
Median follow-up		67.7 mo		42.8 mo		18.1 mo		26.6 mo			ITT: NRE ^c^ PD-L1(+): ~19 mo	
PFS	Median, mo	ITT: 12.3 I/P: 11.6	ITT: 12.3 I/P: 8.3	15.7	11.1	16.6	8.3	23.9	14.7	9.2	ITT ^c^: 13.9 PD-L1(+): 13.8	ITT ^c^: 8.5 PD-L1(+): 7.0
	HR, (95% CI)	ITT: 0.86 (0.73–1.01); *p* = 0.0628 I/P: 0.73 (0.61–0.87); *p* = 0.0004		0.68 (0.58–0.80) *p* < 0.0001		0.51 (0.41–0.64) *p* < 0.001		L + P vs. S: 0.39 (0.32–0.49) *p* < 0.001 L + E vs. S: 0.65 (0.53–0.80) *p* < 0.001			ITT ^c^: 0.69 (0.56–0.84) *p* < 0.0001 PD-L1(+): 0.62 (0.49–0.77) *p* < 0.0001	
OS	Median, mo	ITT: 55.7 I/P: 47.0	ITT: 38.4 I/P: 26.6	45.7	40.1	NR (NE)	NR (22.6-NE)	NR	NR	NR	ITT ^c^: NE PD-L1 (+): NE	ITT ^c^: 37.8 PD-L1 (+): 29.6
	HR, (95% CI)	ITT: 0.72 (0.62–0.85); *p* < 0.0001 I/P: 0.68 (0.58–0.81); *p* < 0.0001		0.73 (0.60–0.88) *p* < 0.001		0.60 (0.40–0.89) ^a^ *p* = 0.001		L + P vs. S: 0.66 (0.49–0.88) *p* = 0.005 L + E vs. S: 1.15 (0.88–1.50) *p* = 0.30			ITT ^c^: 0.67 (0.57–0.79) *p* = 0.0116 PD-L1(+): 0.83 (0.506–1.151) *p* = 0.1301	
ORR (%)		ITT: 39 I/P: 42.0	ITT: 32 I/P: 27.0	60.4	39.6	55.7	27.1	71.0	53.5	36.1	ITT: 52.5 PD-L1(+): 55.9	ITT:27.3 PD-L1(+): 27.2
TRAEs ^b^ (%)		Fatigue: 37 Pruritus: 28 Diarrhea: 27	Diarrhea: 52 Fatigue: 49 PPE: 43	Diarrhea: 54.3 HTN: 44.5 Fatigue: 38.5	Diarrhea: 44.9 HTN: 45.4 Fatigue: 37.9	Diarrhea: 63.8 PPE:40.0 HTN: 34.7	Diarrhea: 47.2 PPE:40.6 HTN: 40.6	Diarrhea: 61.4 HTN: 55.4 Hypothyroidism: 47.2	Diarrhea: 66.5 Stomatitis: 47.6 HTN: 45.6	Diarrhea: 49.4 HTN: 41.5 Stomatitis: 38.5	Diarrhea: 62.2 HTN: 49.5 Fatigue 41.5	Diarrhea: 47.6 Fatigue: 40.1 Nausea: 39.2
FDA approval		I/P: April 16, 2018 [25]		April 19, 2019 [26]		January 22, 2021 [27]		L + P: August 10, 2021 [28]			May 14, 2019 [29]	

^a^ Overall survival analysis of cabozantinib + nivolumab; 98.89% confidence interval was reported based on probability of OS at 12 months. ^b^ Any grade TRAEs are reported. ^c^ Data cut-off date for the updated survival results for ITT population was April 2020. Median follow-up time was not reported. Abbreviations: L + P—lenvatinib plus pembrolizumab; L + E—lenvatinib plus everolimus; S—sunitinib; PFS—progression-free survival; HR—hazard ratio; ORR—objective response rate; CI—confidence interval; FDA—Food and Drug Administration; NRE—not reported; NE—could not be estimated; NR—not reached; ITT—intention-to-treat population; I/P—intermediate/poor risk; TRAEs—treatment-related adverse events; PPE—palmar–plantar erythrodysesthesia; HTN—hypertension.

**Table 2:** Ongoing front-line trials in renal cell carcinoma.

Title	Trial ID	Intervention	Histology	Phase	Sample Size	Primary Endpoint	Status
MK-6482–012	NCT04736706	Pembrolizumab + lenvatinib + belzutifan vs. Pembrolizumab + lenvatinib + quavonlimab vs. Pembrolizumab + lenvatinib	Clear cell RCC	3	1431	PFS and OS	Recruiting
COSMIC 313	NCT03937219	Nivolumab + ipilimumab + cabozantinib vs. Nivolumab + ipilimumab + placebo	Clear cell RCC	3	840	PFS	Active, not recruiting
A Study of AK104 Plus Axitinib in Advanced/Metastatic Clear Cell RCC	NCT05256472	AK104 + axitinib	Clear cell RCC	2	40	ORR	Not yet recruiting
Dendritic Cell Immunotherapy Plus Standard Treatment of Advanced RCC	NCT04203901	Nivolumab + ipilimumab +/− CMN-001 followed by lenvatinib + everolimus	Clear cell RCC	2b	120	OS	Recruiting
PDIGREE Alliance A031704	NCT03793166	Nivolumab + ipilimumab followed by Nivolumab vs. Nivolumab + cabozantinib	Clear cell RCC +/− Sarcomatoid features	3	1046	OS	Recruiting
PIVOT-09	NCT03729245	Bempegaldesleukin + nivolumab vs. Sunitinib or cabozantinib	Clear cell RCC +/− Sarcomatoid features	3	623	ORR and OS	Active, not recruiting
Checkmate 8Y8	NCT03873402	Nivolumab + ipilimumab vs. Nivolumab + placebo	Clear cell RCC +/− Sarcomatoid features	3	437	PFS and ORR	Active, not recruiting
PIVOT IO 011	NCT04540705	Part 1 Nivolumab + Bempegaldesleukin + Axitinib Nivolumab + Bempegaldesleukin + Cabozantinib Part 2 Nivolumab + Bempegaldesleukin + Cabozantinib vs. Nivolumab + Cabozantinib	Clear cell RCC +/− Sarcomatoid features	1/2	251	Safety, DLT, ORR	Recruiting
Study to Evaluate the Efficacy and Safety of Toripalimab in Combination with Axitinib Versus Sunitinib Monotherapy in Advanced RCC	NCT04394975	Toripalimab + axitinib vs. sunitinib	Clear cell RCC +/− Sarcomatoid features	3	380	PFS	Recruiting
PROBE study	NCT04510597	Immunotherapy-based combination +/− cytoreductive nephrectomy	Any RCC histology (Except collecting duct carcinoma)	3	364	OS	Recruiting
MK-3475-03A	NCT04626479	Pembrolizumab/Quavonlimab + Lenvatinib vs. Favezelimab/Pembrolizumab+ Lenvatinib vs. Pembrolizumab + Belzutifan + Lenvatinib vs. Pembrolizumab + Lenvatinib	Any RCC histology	1b/2	390	DLT, AE, ORR	Recruiting
MK-3475-B61/ KEYNOTE-B61	NCT04704219	Pembrolizumab + Lenvatinib	Non-clear cell RCC	2	152	ORR	Active, not recruiting
A Study of Nivolumab In Combination With Cabozantinib in Patients With Non-Clear Cell RCC	NCT03635892	Cabozantinib + nivolumab	Non-clear cell RCC	2	97	ORR	Recruiting
PAXIPEM	NCT05096390	Axitinib +/− pembrolizumab	Type 2 or mixed pRCC	3	72	ORR	Not yet recruiting
The LENKYN Trial	NCT04267120	Pembrolizumab + Lenvatinib	Non-clear cell RCC	2	34	ORR	Recruiting
ALTER-UC-001	NCT05124431	Anlotinib + everolimus	Non-clear cell RCC	2	30	ORR	Not yet recruiting
